# Differential Patterns of Hypoperfusion in Subtypes of Mild Cognitive Impairment

**DOI:** 10.2174/1874440000802010020

**Published:** 2008-05-09

**Authors:** Paolo Caffarra, Caterina Ghetti, Letizia Concari, Annalena Venneri

**Affiliations:** 1 Department of Neuroscience, University of Parma, Italy; 2 Clinical Neuroscience Centre, University of Hull, UK; 3 Fisica Sanitaria, Azienda Ospedaliera Universitaria, Parma, Italy

**Keywords:** Mild Cognitive Impairment, SPECT, cerebral blood flow, Alzheimer’s disease, dementia

## Abstract

In this study the regional cerebral blood flow (rCBF) pattern of three Mild Cognitive Impairment (MCI) subtypes was measured with SPECT in 60 patients (nineteen with an amnestic deficit, sixteen with disexecutive deficits, and twenty five with mild multidomain deficits) and compared with that of 15 healthy matched older adults.

The amnestic MCI subgroup showed significant hypoperfusion in the left hippocampus, parahippocampal gyrus and fronto-parieto-temporal areas. The disexecutive subgroup had significant hypoperfusion of the left superior, medial frontal and cingulate cortex. The multidomain subgroup had similar perfusion deficits to the amnestic subgroup, with an additional deficit in the left posterior cingulate gyrus.

This study found differential patterns of hypoperfusion in MCI subtypes. Since all patients who progressed to dementia converted to probable Alzheimer’s disease, the different rCBF patterns most likely reflect the neuropathological heterogeneity at onset and differences in disease stage.

## INTRODUCTION

The concept of Mild Cognitive Impairment (MCI) was developed to describe a clinical syndrome characterised primarily by memory impairment in relation to age norms, absence of other cognitive abnormalities, normal mental status and preserved functional activities [1]. Patients meeting criteria for MCI have a greater conversion rate to dementia, mostly due to Alzheimer’s disease (AD), than other healthy older adults. MCI is often considered as a clinical condition in which individuals are in a transitional phase between normal and pathological age related decline or as a stage in which the earliest signs of AD become apparent [2-4]. Several studies have stressed the link between MCI and the increased likelihood of developing AD [4-6]. It has become increasingly clearer, however, that the MCI syndrome defines a heterogeneous population and as MCI subjects are more intensively investigated this heterogeneity is gradually more manifest [7]. If MCI with predominant impairment of memory abilities is really a transitional cognitive state that precedes the onset of AD, it is conceivable that other categories of MCI might be found with symptoms involving other cognitive functions with or without relative sparing of memory. This kind of observation should not be unexpected as, although memory deficits are undoubtly one of the earliest hallmarks of AD, this disease is also characterised by heterogeneity of symptoms at onset, and variants to the typical onset profile have been frequently reported [8]. The growing body of clinical evidence prompted a redefinition of the original MCI concept, which is now used to ‘designate an early, but abnormal, state of cognitive impairment’ [9]. MCI subtypes have been proposed on neuropsychological grounds [7, 10]. There was some suggestion that different neuropsychological profiles might align with different diagnostic and aetiological classifications [10], but more recent evidence seems to indicate that conversion to different types of dementia is equally likely in all subtypes [11]. There is no clear structural or functional evidence that these subtypes are the expression of different underlying neuropathological conditions. Structural [12-14] and functional [15-20] studies of individuals meeting the original Petersen *et al’s* criteria for MCI have shown atrophy and reduced function in the mediotemporal cortex, thalamus, posterior cingulate and parietal areas [21]. A recent study which considered the additional contribution of white matter hyperintensities, as an index of cerebrovascular disease, showed that MCI patients with MRI evidence of high vascular burden, additional to hippocampal atrophy, also had a different neuropsychological profile, including working memory and attention deficits [22]. More recent studies have used the redefined diagnostic criteria in patient selection. A study of brain metabolism in the amnestic and multidomain subtypes compared patients who developed AD with those who remained stable [23]. The findings showed that MCI converters had greater hypoperfusion in the parietal and posterior cingulate cortex bilaterally, while non converters had hypometabolism in a more limited region of the frontal cortex. This study also found that metabolic heterogeneity reflected the severity of the memory deficit as measured by the California Verbal Learning Test. A broadly similar pattern of deficit in the posterior cingulate cortex was also observed by a retrospective study which looked at the baseline brain perfusion scans of amnestic, multidomain and non amnestic subtypes converting and non converting to AD [24].

Recently, the comparison of grey matter density in MCI patients, either with a predominantly amnestic pattern or with mild impairment in several cognitive abilities, with the regional grey matter density of a group of healthy controls also produced interesting findings [25]. The results showed bilateral differences in mediotemporal regions and in the middle temporal gyrus in the amnestic MCI subgroup when compared to controls, and greater grey matter loss in the left entorhinal cortex and inferior parietal lobe when compared to the multidomain MCI subgroup. More extensive grey matter loss in the right inferior frontal region, middle temporal gyrus and the superior temporal gyrus bilaterally was present in the multidomain subgroup when compared to the amnestic subgroup. An additional important finding of this study was the presence of a critical difference in the inferior frontal region in both subgroups. This specific regional deficit might be of importance in the clinical presentation of MCI and might flag up early disruption in semantic retrieval which is critical in the clinical manifestation of AD [26-29]. Overall these studies, primarily focused on the amnestic and multidomain subtypes, appear to indicate that the MCI population, even when better characterised in subtypes, still includes a very heterogeneous sample whose difference in perfusion, metabolism or atrophy patterns may simply reflect neuropathological heterogeneity at the onset of the disease. In some instances, the detected differences might simply be a reflection of disease severity. 

This hypothesis was tested in this Single Photon Emission Computed Tomography (SPECT) study of MCI subtypes.

## MATERIALS AND METHODS

### Participants

Sixty individuals meeting current clinical and neuropsychological criteria for a diagnosis of MCI subtypes [1] participated in this SPECT study. These patients were referred to the Centre for Cognitive Disorders at the University of Parma (Italy) because of complaints of cognitive impairment. There were 29 males and 31 females in the group with a mean age of 67.71 years (SD 6.26) and a mean education of 7.11 years (SD 2.81). To exclude the presence of dementia, all individuals underwent a comprehensive clinical and instrumental examination, in line with international published guidelines for the diagnosis of different types of dementia [30-33]. Individuals were included only if there was no neuroimaging evidence of cortical or subcortical vascular lesions on CT or MRI scans and if there was no history of hypertension, diabetes mellitus, transient ischemic attacks or cardiovascular problems. All MCI individuals were examined using an extensive neuropsychological battery, including tests assessing mental status, abstract reasoning, long-term and short-term verbal and visuospatial memory, attention, language, praxis and executive functions. Activities of daily living (ADL) and instrumental activities of daily living (IADL) were also assessed. All MCI participants were normal community dwelling individuals who had no difficulties in carrying out activities of daily living and achieved normal scores on the ADL and IADL scales [34]. Additional informal semistructured clinical questioning highlighted no difficulties in planning and initiating complex functional activities. All tests included in the neuropsychology battery have norms and cut-offs available for the Italian population [35, 36]. 

Based on whether individuals’ neuropsychological scores fell above or below cut-off in individual tests, three different cognitive profiles could be identified corresponding to three subgroups of MCI: amnestic MCI (a-MCI) (N = 19, mean age 65.5 , SD 7.71; mean education 8.9, SD 3.04) with deficits confined to the episodic memory domain, disexecutive MCI (d-MCI) (N=16, mean age 69.62, SD 4.45; mean education 5.81, SD 1.1) with deficits in executive functions only, and multidomain MCI (m-MCI) (N=25, mean age 68.16, SD 5.74; mean education 6.56, SD 2.77) with mild deficits affecting several aspects of their cognitive profile including memory but preservation of functional abilities (see Table **1** for a summary of the neuropsychological profiles of the MCI subgroups). 

A group of 15 healthy age-matched older adults (mean age 65.8, SD 6.62; mean education 8.73, SD 4.26) were included in the study for comparison. These individuals were also assessed with the same neuropsychological battery and clinical criteria as the MCI group to exclude the presence of cognitive decline. Approval for this study was granted by the local ethics committee.

### SPECT Acquisition and Reconstruction

All participants were injected with 740 MBq of ^99m^Tc-HMPAO. One hour after injection they underwent a SPECT scan performed with a dual headed gamma camera (Adac Genesys Vertex) equipped with Fan Beam Long Focus (FBLF) collimators, with a rotation radius of 15-17 cm. The acquisition matrix was 128x128x16 with a voxel size of 4.6 x 4.6 x 4.6 mm, the number of projections were 64, with an acquisition time per projection of 40 seconds. The raw data were reconstructed using a Filter Back Projection technique with a Butterworth 5 order filter and a cut-off frequency of 0.4 cycles cm^-1^. After reconstruction the images were corrected for attenuation with a Chang first order technique and a linear attenuation coefficient of 0.11 cm^-1^. To remove any background signal the brains were masked from the images using a 3D ellipsoid-shaped region of interest (ROI).

To eliminate low intensity background noise and brain edge image artifacts caused by any partial volume effects, images were cut-off twice below the threshold of 30% of the maximum voxel values and then 70% of the mean voxel value [37]. These images were saved in DICOM format and then individually normalised to the cerebellum counts using the ImageJ 1.29x software package (National Institutes of Health, USA). This procedure was followed to ensure that the counts in the cerebellum of each individual were the same prior to any processing, which should in turn result in increased sensitivity and specificity of the analysis [38]. The software ImageJ was also used to convert the images into ANALYZE format for subsequent analysis.

### Image Analysis

Reconstructed images were analysed using the software Statistical Parametric Mapping 2 (SPM2) (Wellcome Department of Imaging Neuroscience, London, UK). Images were normalised into standard stereotactic space. A 12 parameter affine transformation was used when normalising images to the standard SPECT template provided in SPM2. Normalised images were then smoothed with an isotropic Gaussian kernel set at 10 mm of full width at half maximum (FWHM). Between-group differences in regional cerebral blood flow (rCBF) were assessed on a voxel-by-voxel basis using independent sample t-test or ANCOVA, with education as a covariate when appropriate.

## RESULTS

There were no significant differences between the MCI and control samples for age and education (t_73_ 1.04, n.s. and t_73_ -1.779, n.s. respectively). No significant differences were found between the age and the education level of the a-MCI subgroup and the control group (t_32_ -0.109, n.s. and t_32_ 0.170, n.s., respectively) and between the age and the education level of the m-MCI subgroup and the control group (t_38_ 1.187, n.s. and t_38_ -1.957, n.s., respectively). Controls and d-MCI did not differ in age (t_29_ 1.897, n.s.), but their level of education was significantly different (t_29_ -2.647, p < 0.05).

The SPECT images of the three MCI subgroups were, therefore, compared using independent sample t-test or ANCOVA, with education as a covariate in the case of the controls/d-MCI subgroup comparison. Height threshold was set at 0.05 (FWE corrected).

### a-MCI Versus Older Adult Controls

**Fig. (1):**
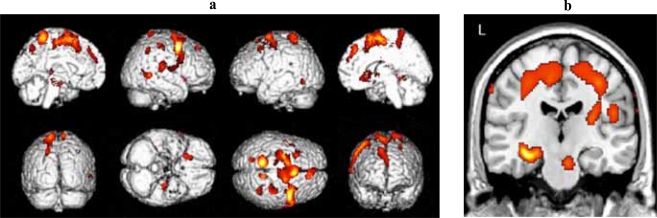
**(a)** Three-dimensional standard rendering of the brain showing the areas of reduced cerebral blood flow in the amnestic MCI group. **(b)** Coronal section showing significant hypoperfusion in the hippocampus.

Significant clusters of reduced blood flow were detected in the left hippocampus and parahippocampus, and in regions of the frontal, temporal and parietal lobes bilaterally (see Table **2a** for detailed listing of areas and Fig. **1**). There also was significant hypoperfusion in the region of the left and right thalamic nuclei and of the left lentiform nucleus.

### d-MCI Versus Older Adult Controls

**Fig. (2):**
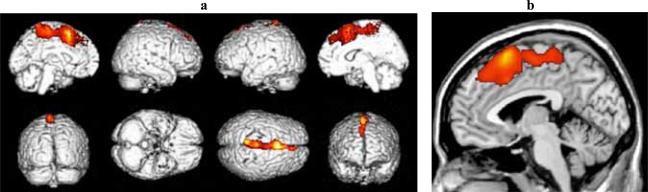
**(a)** Three-dimensional standard rendering of the brain showing the areas of reduced cerebral blood flow in the disexecutive MCI group. **(b)** Sagittal section showing significant hypoperfusion in the anterior cingulate cortex.

Significant clusters of reduced blood flow were found in the left superior frontal and medial frontal gyri and in the anterior cingulate cortex (see Fig. **2** and Table **2b**).

### m-MCI Versus Older Adult Controls

**Fig. (3):**
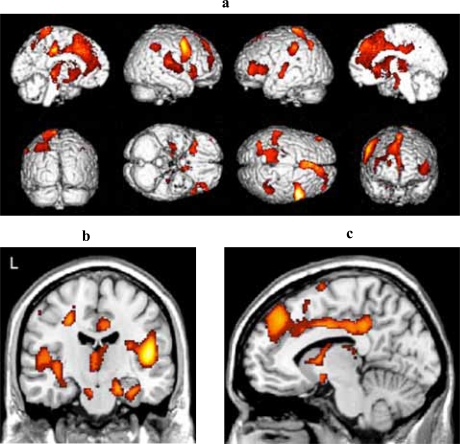
**(a) **Three-dimensional standard rendering of the brain showing the areas of reduced cerebral blood flow in the multidomain MCI group.** (b)** Coronal section showing significant hypoperfusion in mediotemporal regions. **(c)** Sagittal section showing significant hypoperfusion in the anterior and posterior cingulate cortex.

Fig. (**3a**) shows the areas of reduced blood flow. Significant reduction in blood flow were found in the mediotemporal regions bilaterally (right hippocampus, left and right parahippocampal gyrus) (Fig. **3b**), in the lingual gyrus bilaterally, in several structures of the frontal, parietal and temporal cortex bilaterally, and in the left cigulate cortex (see Table **2c** for detailed listing and Fig. **3b**). There also were some areas of significant hypoperfusion in the region of the right and left caudate nucleus.

### Clinical Follow-Up

Monitoring of these patients in the clinic is still ongoing. At present, follow-up time ranges between 12 and 24 months. None of the a-MCI people showed signs of conversion to AD, 42% in the d-MCI group converted to dementia during the follow up period, and although with an atypical onset, when reviewed after two years these patients met clinical criteria for AD [30]. Four patients in the m-MCI group (n= 25) could not be traced for follow–up. Among those remaining, 55% had progressed to AD when reviewed after 24 months. None of the controls developed any change in cognitive functioning during the same follow-up period. Although sample size was small and the validity of a statistical comparison questionable, an attempt was made to see whether there was any difference in rCBF patterns between the stable and converter MCI patients. No significant differences were found. Other comparisons taking gender and education levels into account also showed no significant differences.

## Discussion

This study has shown that cognitive heterogeneity in patients with MCI is associated with specific patterns of cerebral hypoperfusion when compared with healthy older adult controls. The a-MCI group, who included patients with selective verbal episodic memory deficits, had a perfusion profile similar to that found in rCBF or brain metabolism studies of patients in the early stages of AD [39, 40]. In the Desgranges *et al*. study, a significant correlation was found between metabolism values in left mediotemporal regions (including the parahippocampal gyrus and frontal regions) and verbal episodic memory scores in patients with mild to moderate AD. The regions showing hypoperfusion were those which are part of a distributed memory network and these brain areas often show significant activation in response to paradigms involving memory encoding and retrieval in functional neuroimaging studies of memory [41-45].

Similarly to other published neuroimaging studies of MCI patients, rCBF dysfunction was not exclusively confined to mediotemporal regions but extended to the associative fronto-temporal-parietal cortices [17-20, 46]. No significant hypoperfusion in the posterior cingulate cortex was found in the a-MCI sample; there was, however, a significant difference in perfusion in the precuneus (BA 7), a structure adjacent to the posterior cingulate region (BA 31) reported as dysfunctional in most of the published studies. A deficit in this area has been indicated as a useful functional marker of disease progression in MCI and reported in other studies (e.g. [16-20]). It is possible that the patients imaged in the present study were less severe and less heterogeneous in terms of memory impairment (all patients in this subgroup showed deficits in delayed verbal recall). The lack of perfusion deficit in the posterior cingulate cortex in the amnestic subgroup might explain why these patients remained fairly stable over the observation period. Other studies have, in fact, shown major functional deficits in the posterior cingulate area only in those a-MCI who converted to AD but not in those who did not [23, 24].

The a-MCI subgroup had extensive perfusion differences from controls in fronto-temporo-parietal regions. This significant hypoperfusion was broadly asymptomatic and did not severely affect cognitive performance in functions associated with these cortical regions. Overall, the pattern of hypoperfusion in the a-MCI subgroup resembled that of early AD and the data provide some support to the notion that the a-MCI condition might be prodromal to the development of Alzheimer type dementia. 

The pattern of hypoperfusion detected in the disexecutive subgroup was very different from that of the a-MCI. Significant rCBF reduction was confined to the frontal cortex and involved the anterior cingulate gyrus. All the patients in the d-MCI group had deficits only in attention and executive tasks and an otherwise normal neuropsychological profile. An association between failure to achieve good performance on the Wisconsin Card Sorting test and/or the Stroop test and damage to the frontal lobe has been repeatedly reported in the neuropsychological literature [47-49]. Some involvement of the anterior cingulate cortex is the primary finding in neuroimaging studies which have used a Stroop paradigm [50, 51]. A recent study has also demonstrated that different groups of patients (MCI, AD, Frontal Lobe Dementia), whose common characteristics was failure on the Wisconsin Card Sorting test, showed a correlation between significant rCBF reduction in the rostrodorsal prefrontal cortex and the number of perseverative errors made by the patients [52]. A focal neuropsychological profile was consistent with the regionally confined rCBF deficit. The atypical onset cognitive profile and related regional blood flow deficit detected in the d-MCI subgroup does not appear to be of any aetiological value, as all the patients who converted to dementia in this subgroup met clinical criteria for a diagnosis of probable AD and not those for frontotemporal dementia.

The rCBF difference between controls and m-MCI patients was similar to that of the a-MCI subgroup. Significantly reduced perfusion was present in fronto-temporal-parietal regions as well as the hippocampus and parahippocampus. There was, however, significant hypoperfusion in the posterior cingulate cortex, a finding which is in line with those of other similar studies [16-20]. The presence of significant deficits in the posterior cingulate gyrus might indicate a more advanced disease stage and justify the higher rate of conversion to dementia in this subgroup. The value of deficits in the posterior cingulate cortex as predictor of conversion from MCI to AD has been highlighted by other functional and structural neuroimaging studies [23, 24]. A significant difference from controls in cingulate cortex is also frequently found in early AD [18]. Significant perfusion deficits in this posterior limbic region might indicate that the patients with mild impairments in several cognitive domains, although still retaining functional abilities, pathophysiologically are most likely in a more advanced disease stage and closer to transition from MCI to early AD. The findings in the m-MCI subgroup are also in agreement with the evidence from a recent PET study which showed that a reduction in regional glucose metabolism rate at rest in the inferior parietal cortex predicted later conversion to AD [53]. Overall, the present study highlighted regions of dysfunction in the m-MCI subgroup which were similar to those where structural deficits have been detected with voxel based morphometry in individuals with this MCI subtype [25]. 

In conclusion, this study has demonstrated that the neuropsychological profiles of MCI subgroups are most likely the expression of the neuropathological heterogeneity which characterises AD in the early stages. There is no evidence in these data that the neuropsychological profiles of different MCI subgroups are strongly predictive of a different aetiology, as all the patients who converted to dementia in this sample met clinical criteria for probable AD and not those for other types of degenerative dementia. A longer period of observation and a much larger sample might be needed to address aetiological issues. The data seem to indicate that the rCBF patterns of dysfunction, although not useful in differential diagnosis, might provide functional indices which can be clinically relevant in the patients’ clinical assessment. The findings also lend some support to recent views that the diagnostic definitions of MCI and AD, as characterised by current clinical criteria, need to be refined to reliably identify those patients who are in the earliest stages of the disease [54].

Detailing the neuropsychological profile and brain blood flow/metabolism pattern of patients meeting criteria for MCI, therefore, might improve clinical assessment in this population, with implications for the adoption of appropriate intervention and monitoring at a stage when these patients and their families might benefit most from treatment.

## Figures and Tables

**Table 1.** Mean (and Standard Deviation) Scores Achieved by MCI and Controls on the Neuropsychological Tests Included in the Standard Cognitive Assessment Battery

Test	Amnestic MCI	Disexecutive MCI	Multidomain MCI	Controls	Cut-off Value (*)
Mini Mental State Exam	27.15 (1.3)	27.12 (1.25)	25.24 (0.87)	29.2 (1.08)	< 24.00
Progressive Matrices (PM47)	27.68 (2.51)	21.88 (4.61)	24.43 (4.32)	28.2 (4.16)	< 17.50
Story recall	4.34 (3.27)	7.53 (0.71)	3.12 (2.46)	13.1 (0.38)	< 7.50
Rey -15 words (immediate recall)	29.15 (10.87)	30.08 (5.52)	21.52 (8.77)	41.2 (11.6)	< 28.53
Rey -15 words (delayed recall)	4.52 (3.61)	5.81 (1.37)	2.64 (1.97)	8.2 (3.70)	< 4.69
Rey complex figure * *(copy)	31.42 (3.23)	29.25 (2.29)	27.15 (2.90)	32.4 (2.50)	< 28.87
Rey complex figure (delayed recall)	15.18 (7.49)	11.31 (2.70)	3.92 (4.18)	16.5 (5.63)	< 9.46
Letter fluency	29.68 (11.36)	20.72 (7.21)	16.75 (9.83)	24.8 (7.33)	< 17.35
Category fluency	33.36 (10.15)	27.68 (3.78)	20.80 (7.90)	34 (6.51)	< 24.00
Stroop test (time interf)	22.10 (8.55)	40.59 (6.71)	44.3 (24.74)	22.16 (5.43)	> 36.92 (**)
WCST (categories)	4.63 (1.16)	1.87 (0.95)	2.64 (1.43)	5.4 (0.82)	< 2.00
WCST (no of persev errors)	2.52 (2.34)	11.0 (6.0)	8.60 (5.37)	2 (1.13)	> 6.41 (**)
Digit cancellation test	47.57 (7.14)	43.5 (5.2)	39.96 (9.0)	52.8 (5.97)	< 30.00

*Cut-off values refer to published norms for the Italian population and indicate the score below which performance falls in the abnormal range. **In these measures cut-off values indicate the score above which performance falls in the abnormal range.

**Table 2. Areas of Reduced rCBF (and Corresponding Brodmann’s Areas) in (a) the Amnestic MCI, (b) the Disexecutive MCI, and (c) the Multidomain MCI Subjects, when Compared with Healthy Matched Controls:** (a) Amnestic MCI

Brain Region (BA)	Cluster Size	Cluster Level p Value (corrected)	Z Value	Talairach Coordinates x y z
Left hippocampus	3349	0.000	Inf.	-32 -18 -13
Left parahippocampal gyrus (BA 28, 34)		0.000	Inf.	-24 -22 -11
		0.000	6.06	-18 -10 -14
Left precentral gyrus (BA 44)		0.000	6.54	-46 2 9
Right middle frontal gyrus (BA 6)	32494	0.000	Inf.	48 9 55
		0.000	Inf.	51 6 46
Left postcentral gyrus (BA 5)		0.000	7.72	-14 -42**63
Left middle frontal gyrus (BA 9)	97	0.002	5.81	-51 27 30
Right superior frontal gyrus (BA 10)	40	0.007	5.76	32 56 23
Right middle frontal gyrus (BA 10)		0.007	5.33	38 53 18
Left lingual gyrus (BA 18)	436	0.000	5.68	-16 -88 -14
		0.000	5.12	-8 -73 -15
Left fusiform gyrus (BA 18)		0.000	5.09	-26 -86 -16
Left inferior frontal gyrus (BA 47)	476	0.000	5.49	-18 32 -16
Left middle frontal gyrus (BA 47)		0.000	5.38	-26 35 -8
Right precuneus (BA 7)	82	0.002	5.45	4 -69 55
Left thalamus	105	0.001	5.33	-10 -7 15
Right middle temporal gyrus (BA 39)	31	0.010	5.30	51 -71 24
Left middle temporal gyrus (BA 21)	37	0.008	5.21	-62 -2 -24
Left lentiform nucleus	164	0.000	5.17	-24 12 -2
Left claustrum		0.000	4.50	-32 10 9
Right fusiform gyrus (BA 37)	80	0.002	5.07	50 -59 -21
Right pulvinar	77	0.003	4.80	4 -33 7
Right precuneus (BA 7)	21	0.014	4.79	20 -79 45
Right cuneus (BA 18)		0.014	4.76	6 -93 8

**Table:** (b) Disexecutive MCI

Brain Region (BA)	Cluster Size	Cluster Level p Value (corrected)	Z Value	Talairach Coordinates xyz
Left superior frontal gyrus (BA 6)	7096	0.001	5.94	-4 14 59
Left cingulate gyrus (BA 32)		0.001	4.94	-2 14 42
Left medial frontal gyrus (BA 6)		0.001	4.40	-3 -21 55

**Table:** (c) Multidomain MCI

Brain Region (BA)	Cluster Size	Cluster Level p Value (corrected)	Z Value	Talairach Coordinates xyz
Right middle frontal gyrus (BA 6)	1198	0.000	7.62	57 8 40
		0.000	7.47	51 6 46
Right inferior frontal gyrus (BA 9)		0.000	6.61	59 15 27
Right insula	1443	0.000	7.43	50 -23 16
	1538	0.000	7.39	46 -17 10
		0.000	6.29	32 21 1
Right caudate head		0.000	6.22	20 23 1
Right caudate body		0.000	6.05	12 5 13
		0.000	5.91	30 -26 14
Left cingulate gyrus (BA 31, 32)	4057	0.000	7.03	-12 -23 40
		0.000	6.79	-1 29 34
Right medial frontal gyrus (BA 8)		0.000	6.00	8 41 37
Right parahippocampal gyrus (BA 28, 37)	243	0.000	5.98	18 -16 -16
		0.000	4.77	16 -12 -8
Right hippocampus		0.000	5.78	32 -15 -21
Left superior frontal gyrus (BA 8)	116	0.001	5.88	-30 30 54
Left inferior parietal lobule (BA 7)	1024	0.000	5.47	-34 -60 49
Right superior frontal gyrus (BA 10)	92	0.002	5.61	42 52 16
		0.002	5.08	30 57 19
		0.002	4.86	42 53 7
Right inferior parietal lobule (BA 40)	441	0.000	5.59	42 -43 43
Left lingual gyrus (BA 18)	179	0.000	5.55	-16 -80 -9
Left parahippocampal gyrus (BA 28)	1165	0.000	5.55	-18 -26 -7
Right pulvinar		0.000	5.51	2 -27 5
Left superior temporal gyrus (BA 22)		0.000	5.41	-50 -12 -3
Right lingual gyrus (BA 18)	67	0.004	5.32	18 -78 -10
Left inferior frontal gyrus (BA 10, 45)	621	0.000	5.30	-40 46 2
		0.000	5.23	-50 18 3
Left middle frontal lobe (BA 9)	64	0.005	5.29	-50 27 32
Right postcentral gyrus (BA 2)	27	0.013	5.18	65 -23 40
Left caudate body	24	0.015	5.06	-18 7 20
Left postcentral gyrus (BA 2)	408	0.000	5.02	-51 -29 36
Left inferior parietal lobule (BA 40)		0.000	4.93	-57 -31 42
		0.000	4.92	-38 -33 46
